# A Cyanotic Infant: Infrequent Presentation of Cow’s Milk Protein Allergy

**DOI:** 10.7759/cureus.21678

**Published:** 2022-01-28

**Authors:** Paula García Sánchez, Guillermo Santos Simarro, Mercedes Sampredro Martín, Laura Valladares Salado, Lucía Escolano Taravillo

**Affiliations:** 1 Emergency Department, La Paz University Hospital, Madrid, ESP; 2 Pediatrics, La Paz University Hospital, Madrid, ESP; 3 Pediatric Infectious Disease Department, La Paz University Hospital, Madrid, ESP

**Keywords:** cow's mil protein allergy, cyanosis, pfies, hydrolyzed formula, methemoglobinemia

## Abstract

We report the case of a three-month-old boy who presented with poor weight gain, loose stools, and poor oral intake for three weeks. Physical examination revealed a pale infant with abdominal distension and cyanosis. Oxygen saturation was normal, but the laboratory showed important methemoglobinemia. The diagnosis of FPIES (food protein-induced enterocolitis syndrome) in the context of cow’s milk protein allergy (CMPA) was suspected. Although CMPA is a common condition encountered in small children, chronic forms of FPIES can be difficult to diagnose. Maintaining clinical suspicion about the potential association between methemoglobinemia and gastrointestinal symptoms can lead to prompt recognition and intervention.

## Introduction

Cow’s milk protein allergy (CMPA) is a common condition seen in young children, particularly in the first year of life [1], with an incidence estimated as 2% to 7.5% [2]. CMPA is classified into immunoglobulin E (IgE)- or non-IgE-mediated reactions, which vary in terms of clinical manifestations, diagnostic evaluation, and prognosis. Non-IgE mediated forms include a range of symptoms predominantly affecting the gastrointestinal system, with varying severity [1,2]. Chronic forms of FPIEs occur mainly in children under four months of age after repeated ingestion of cow’s milk protein (CMP), characterized by intermittent vomiting, chronic diarrhea, and weight loss [1]. Blood tests may show anemia, hypoalbuminemia, thrombocytosis, leukocytosis with left shift and eosinophilia, dehydration, metabolic acidosis, or methemoglobinemia [1]. Treatment consists of the elimination of CMP from the diet [1].

## Case presentation

In February 2021, a three-month-old boy presented to La Paz Hospital Emergency Department (Madrid, Spain) with a history of poor weight gain, loose stools, and poor oral intake for three weeks. He was slightly irritable, with no vomiting or other symptoms. The patient’s past medical history was unremarkable, and he was exclusively fed cow’s milk formula from birth.

**Figure 1:**
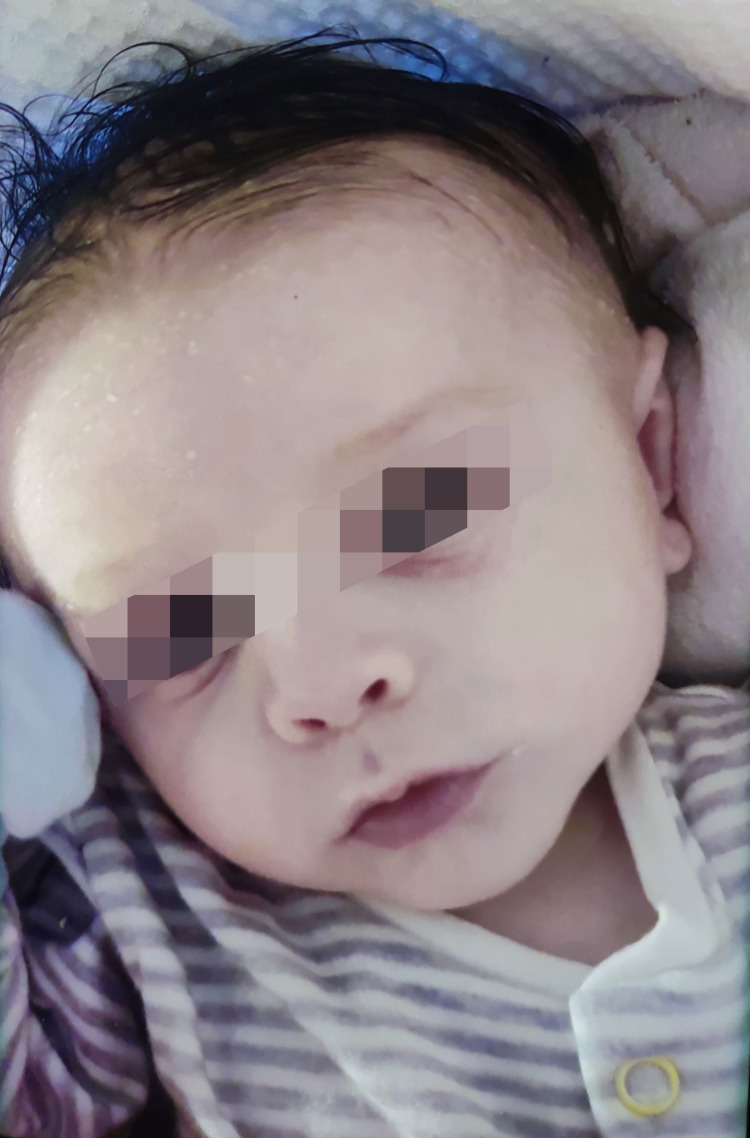
Marked cutaneous pallor with cyanosis of the lips

On admission, the patient was active, alert, and interactive, but his skin was pale with cyanotic lips (Figure 1).

**Figure 2:**
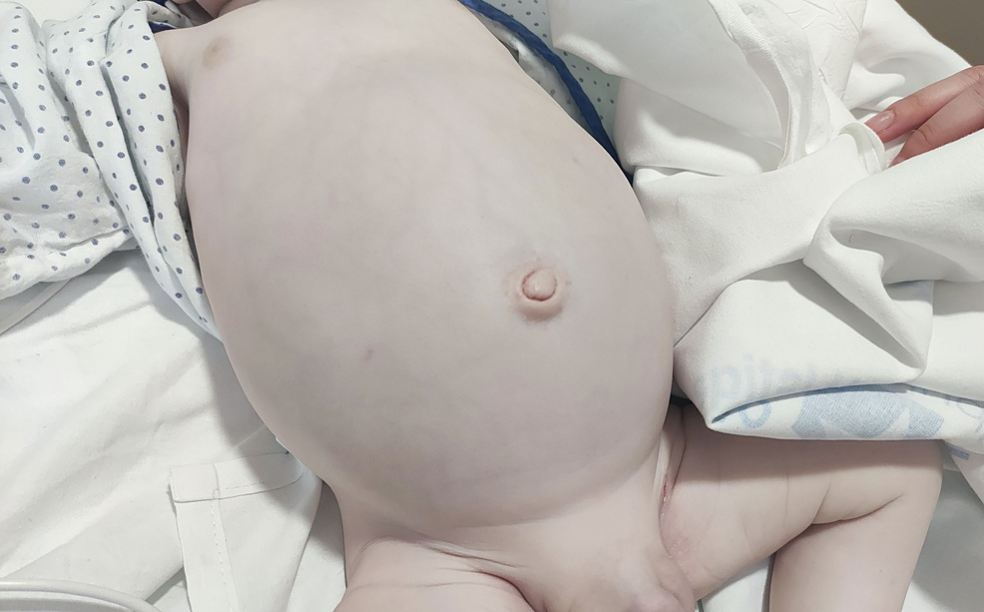
Generalized cutaneous pallor with significant abdominal distension

The abdomen was significantly distended and soft, with normal bowel sounds (Figure 2).

Vitals signs showed heart rate 147/min, respiratory rate 25/min, blood pressure 90/56 mmHg, temperature 36.9ºC, and oxygen saturation 98%. Weight was 5.050 kg (9^th ^percentile). Complete blood count, blood culture, and urine culture were drawn, and intravenous fluid therapy was administered. Bloodwork drawn at admission showed white blood cells 17.3 (neutrophils 51%), pH 7.38, bicarbonate 19.5 mmol/L, base deficit -6.3, lactate 1.8 mmol/L, hemoglobin 10.7 g/dL, and methemoglobin 17.6%. Serum electrolytes, renal and liver function tests were normal.

Given the clinical presentation and lab results, a differential diagnosis of FPIES (food protein-induced enterocolitis syndrome) in the context of cow’s milk protein allergy (CMPA) was suspected. Oxygen therapy at 1 lpm was started, he was admitted to the hospital and started a hydrolyzed formula. Methemoglobin levels did not rise to levels typically requiring treatment, so methylene blue therapy was not started. Other causes of methemoglobinemia in infants, such as the use of gels containing benzocaine, were excluded.

After initiation of oxygen therapy and withdrawal of cow's milk protein, blood methemoglobin concentration was measured several times; and a gradual decrease was shown during follow-up. The methemoglobin level decreased from 17.6% to 7% at four hours, and at 12 hours was only 1.5%. He was discharged home after 36 hours with an excellent general condition and adequate oral tolerance of the hydrolyzed formula. One week later, an ambulatory control was performed with normal methemoglobin levels. The symptoms resolved with complete avoidance of cow’s milk.

## Discussion

Cow’s milk protein allergy is common in children in the first year of life. There are two forms: IgE-mediated and non-IgE mediated reactions, and non-IgE mediated reactions can be acute or chronic.

International consensus guidelines on diagnosis and management of FPIES were published in 2017 [3]. These guidelines establish the diagnostic criteria for patients with acute or chronic FPIES. The diagnosis of acute FPIES requires that a patient meets the major criterion and ≥ 3 minor criteria summarized in Table 1 [1,3].

**Table 1:** Diagnostic criteria for acute FPIEs

Major Criterion	Occurrence of vomiting within the first one to four hours after consumption without the classic symptoms of IgE-mediated allergy
Minor criteria	A second episode of repetitive vomiting following intake of cow’s milk. Repetitive vomiting episode within 1-4 hours following consumption of a different food. Extreme lethargy with any suspected reaction. Marked pallor with any suspected reaction. Need for emergency department visit with any suspected reaction. Need for intravenous fluid support with any suspected reaction. Diarrhea 24 hours after ingestion. Hypotension. Hypothermia.

On the other hand, chronic forms of FPIEs are less well characterized, so the diagnosis may be more challenging and may even be delayed. The presence of intermitting vomiting, persistent non-bloody or bloody diarrhea, poor weight gain, and abnormal blood tests (anemia, hypoalbuminemia, thrombocytosis, leukocytosis with left shift, eosinophilia, metabolic acidosis, or methemoglobinemia) after daily ingestion of CMP should make us suspect this entity. Diagnosis of chronic FPIES is based on the resolution of symptoms within days of eliminating the CMP and the acute recurrence of symptoms when it is reintroduced, although oral food challenge is rarely needed to confirm the diagnosis [3].

Methemoglobin is an altered state of hemoglobin in which the heme iron is oxidized from the ferrous (Fe2+) to the ferric (Fe3+) form. As a result, methemoglobin is insufficient for carrying oxygen, leading to cyanosis [4]. The blood concentration of methemoglobin is normally between 0% and 2%. Treatment with methylene blue should be considered when the concentration rises above 20% in symptomatic patients and 30% in asymptomatic patients, although the decision to treat may be made at lower concentrations if symptoms persist or are severe. 

Our patient presented with clinical and laboratories findings consistent with chronic FPIES. An adequate anamnesis, clinical presentation, and laboratory findings were key to suspect this diagnosis. Clinically, this is not the most common manifestation, although severe methemoglobinemia with acute intestinal inflammation has been previously described in FPIES [5-7]. There is no exact cause of methemoglobinemia in FPIES, but the result is severe intestinal inflammation and decreased catalase activity, resulting in increased intestinal nitrites and increased heme molecule oxidation, which contributes to methemoglobinemia [5,8]. Our patient did not require a confirmatory oral food challenge as he had clinical and analytical findings characteristic of the disease, and the symptoms resolved after removing cow’s milk from the diet.

## Conclusions

We report a chronic presentation of FPIEs that initially went unnoticed, leading to a situation of poor weight gain, poor oral intake, abdominal distension with diarrhea, cyanosis, and methemoglobinemia. The improvement of symptoms following the withdrawal of cow’s milk from the diet was essential to establish the diagnosis. Methemoglobinemia in children is usually due to other more frequent causes, but this entity should be kept in mind. Doctors should maintain a high level of suspicion at initial presentation to avoid delay in treatment and progression to a chronic state, which might lead to complications.
